# Cuf2 Is a Novel Meiosis-Specific Regulatory Factor of Meiosis Maturation

**DOI:** 10.1371/journal.pone.0036338

**Published:** 2012-04-27

**Authors:** Raphael Ioannoni, Jude Beaudoin, Luis Lopez-Maury, Sandra Codlin, Jurg Bahler, Simon Labbe

**Affiliations:** 1 Département de Biochimie, Faculté de Médecine et des Sciences de la Santé Université de Sherbrooke, Sherbrooke, Québec, Canada; 2 Department of Genetics, Evolution and Environment, University College London, London, United Kingdom; University of Cambridge, United Kingdom

## Abstract

**Background:**

Meiosis is the specialized form of the cell cycle by which diploid cells produce the haploid gametes required for sexual reproduction. Initiation and progression through meiosis requires that the expression of the meiotic genes is precisely controlled so as to provide the correct gene products at the correct times. During meiosis, four temporal gene clusters are either induced or repressed by a cascade of transcription factors.

**Principal Findings:**

In this report a novel copper-fist-type regulator, Cuf2, is shown to be expressed exclusively during meiosis. The expression profile of the *cuf2^+^* mRNA revealed that it was induced during middle-phase meiosis. Both *cuf2^+^* mRNA and protein levels are unregulated by copper addition or starvation. The transcription of *cuf2^+^* required the presence of a functional *mei4^+^* gene encoding a key transcription factor that activates the expression of numerous middle meiotic genes. Microscopic analyses of cells expressing a functional Cuf2-GFP protein revealed that Cuf2 co-localized with both homologous chromosomes and sister chromatids during the meiotic divisions. Cells lacking Cuf2 showed an elevated and sustained expression of several of the middle meiotic genes that persisted even during late meiosis. Moreover, cells carrying disrupted *cuf2Δ*/*cuf2Δ* alleles displayed an abnormal morphology of the forespore membranes and a dramatic reduction of spore viability.

**Significance:**

Collectively, the results revealed that Cuf2 functions in the timely repression of the middle-phase genes during meiotic differentiation.

## Introduction

Meiosis is a specialized type of cell division by which sexually reproducing diploid organisms generate haploid gametes [1]. Gametogenesis starts with the pre-meiotic S-phase during which the DNA is replicated, thereby generating pairs of homologous chromosomes. Subsequently, these homologous chromosomes are subjected to genetic recombination, a process which is known to contribute to phenotypic diversity within a given population [2]. Homologous chromosomes and sister chromatids are then successively segregated, generating four haploid sets of chromosomes that are inheritable to the next generation. In the terminal stage of meiosis, a differentiation program is induced in order to generate four mature gametes ready for fertilization. Whereas these meiotic hallmarks have been extensively characterized, the molecular mechanisms that control meiotic progression and gamete maturation in higher eukaryotes remain less well understood.

Haploid gametes are present in very small numbers in most mammals, making molecular studies that require substantial amounts of material very arduous [3]. An additional difficulty comes from the fact that both animal models and tissue co-culture cells are not easy to synchronize with respect to their entry into meiosis [4], [5]. Consequently, the use of model organisms has turned out to be a practical solution for the study of the molecular mechanisms that initiate and control meiosis [6], [7]. Among these models, the fission yeast *Schizosaccharomyces pombe* has become particularly attractive for the study of the key molecular aspects of meiosis, from its initiation through to the generation of mature haploid cells [8]. *S. pombe* cells essentially undergo meiosis in a manner analogous to that of the germ line cells in higher eukaryotes, except that the gametes differentiate into spores that are enclosed in an ascus. Each ascus contains four haploid spores that are highly resistant to adverse environmental conditions [9].

Nitrogen availability triggers the decision to follow either the mitotic or the meiotic pathway in *S. pombe*. Under nitrogen-rich conditions, cells grow mitotically because the Pat1 kinase inhibits the initiation of meiosis by phosphorylating both the transcription factor Ste11 and the meiotic inducer Mei2 [10]. Conversely, under nitrogen-starved conditions, the Ste11 transcription factor becomes active and induces the expression of the mating type loci [11]. As a consequence, haploid cells of the opposite mating types conjugate, forming diploid zygotes. A cascade of transcription factors then enables the expression of *mei3* ^+^, which encodes for an inhibitor of the Pat1 kinase [12]. Once, Mei3 inhibits Pat1, the latter becomes unable to phosphorylate its target proteins, including Ste11 and Mei2. As an active Ste11 fosters *mei2* ^+^ expression, unphosphorylated Mei2 accumulates and triggers the initiation of zygotic meiosis [10]. Zygotes can be returned to a nitrogen-rich medium before commitment to meiosis and will resume vegetative growth, forming colonies of diploid cells. Conveniently, these cells will undergo azygotic meiosis in response to a nitrogen starvation shock in a more synchronous manner than zygotic meiosis [13]. The *pat1-114* allele encodes a thermosensitive version of the Pat1 kinase. Consequently, cells harboring the *pat1-114* mutation show temperature-sensitive growth and undergo meiosis and sporulation at the restrictive temperature (34°C), thus bypassing the Mei3-dependent inactivation pathway of Pat1. The advantage of *pat1*-induced meiosis is that it is more synchronous than azygotic meiosis [13].

In fission yeast, meiosis progression is driven by an extensive gene expression program during which the expression of several genes is either induced or repressed [14]. Transcriptional profiles have defined four successive waves of gene expression that are mainly controlled by key meiosis-specific regulators [15]. First, nitrogen starvation triggers the activation of Ste11, which in turn activates the expression of the nutrient-responsive genes so as to initiate meiosis [11]. The expression of the nutrient-responsive genes is subsequently repressed by the transcription factor Rep1, which in turn activates the expression of several early meiotic genes [15]. Once chromosome pairing and homologous recombination have been completed, the transcription factor Mei4 activates the expression of the middle meiotic genes and represses that of the early genes [16], [17]. Rep1 and Mei4 function in both the activation and the repression of gene expression. Although the transcription factors Atf21, Atf31 and Rsv2 activate the expression of the late meiotic genes after the meiotic divisions, it is still unclear how the middle genes are repressed at the end of the middle meiotic phase, a step that precedes late meiosis [15].

When the previously identified potential meiotic transcriptional regulators were examined, one, denoted Cuf2 (*SPCC584.02*) was found to be a putative copper-fist-like transcription factor whose expression profile exhibited a peak that coincided with both the meiotic divisions and the forespore membrane formation (FSM) [14]. Based on sequence homologies with other metalloregulatory transcription factors, it is predicted that the amino-terminal residues 1 to 40 of Cuf2 contains a Zn^2+^ module that is required for DNA minor groove binding [18]. The Cuf2 protein exhibits 42% sequence identity with the N-terminal 61 amino acid residues of the copper-fist-like transcription factor Cuf1 [19], [20]. In *S. pombe*, Cuf1 activates transcription of genes that encode for Ctr copper transporters (Ctr4, Ctr5 and Ctr6) that are involved in copper acquisition [19], [21]–[23]. Structurally, the similarity between the Cuf1 and Cuf2 proteins resides exclusively within the amino-terminal 61-residue segment of the two proteins. As is suggested for Cuf1-related transcription factors, residues 41–60 of Cuf2 that display high similarity with the residues 41–61 of Cuf1 may be required in order to allow the transcription factor to make contact with the major groove of DNA [19], [24].

To gain further insight into the role of Cuf2, its expression profile was characterized. It was discovered that Cuf2 was strictly expressed during meiosis. The expression of *cuf2^+^* required the presence of a functional *mei4^+^* gene, which encodes a key regulator of several middle-phase meiotic genes. Microscopic analyses revealed that a functional Cuf2-GFP protein co-localized with the chromosomes that underwent late anaphase I, metaphase II, anaphase II (early and late) and FSM formation. Furthermore, *pan*-*S. pombe* microarray analysis revealed that 247 genes were up-regulated in a *cuf2Δ/cuf2Δ* disruption strain. In the absence of Cuf2, forespore membrane formation was abnormal and spore viability was significantly reduced. Taken together, the results of these studies revealed that Cuf2 is a meiosis-specific transcriptional regulator that is required for the down-regulation of a large set of middle-phase meiotic genes during meiotic development.

## Results

### *S. pombe* Cuf2 protein

**Figure 1:**
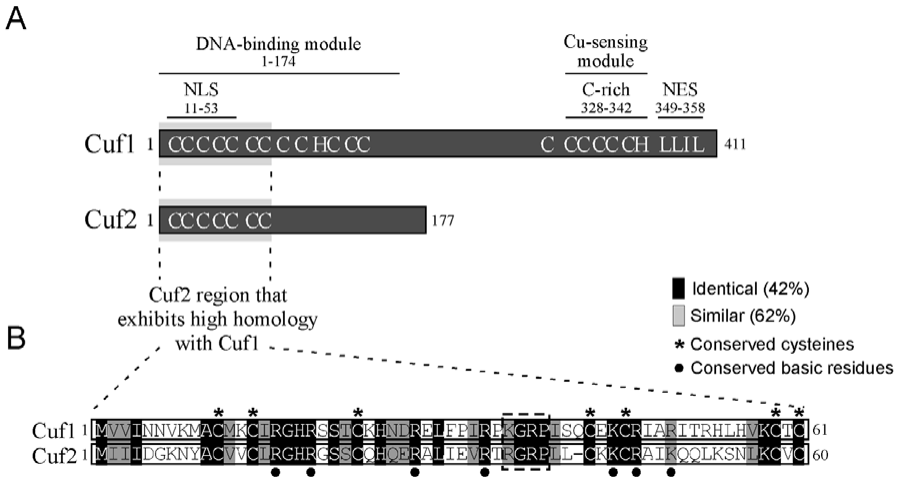
Comparison of Cuf2 with the *S. pombe* copper metalloregulatory transcription factor Cuf1. *A*, Schematic representations of Cuf1 and Cuf2. The amino acid sequences of both proteins are numbered relative to their initiator codons. The locations of the domains required for Cuf1 function are indicated, including the N-terminal nuclear localization signal (NLS) (11–53) that is located within the DNA-binding module (1–174), the C-terminal Cu-sensing module (C-rich) (328–342) and the C-terminal nuclear export signal (NES) (349–358). The positions of some of the Cys (C) and His (H) residues within both Cuf1 and Cuf2 are also indicated. *B*, Amino acid alignment of the N-terminal 61 amino acid residues of Cuf1 with the N-terminal 60-residue segment of Cuf2. The black boxes indicate identical amino acids, and the gray boxes indicate amino acids that are similar between Cuf2 and Cuf1. The asterisks highlight the 7 Cys residues that are conserved between Cuf2 and Cuf1. The indicated N-terminal 1–60 amino acid region of Cuf2 exhibits 42% sequence identity and 62% sequence similarity with the N-terminal 61 amino acids of Cuf1 that are part of its DNA-binding domain.

Analysis of genomic DNA sequences from the *S. pombe* Genome Project revealed an open reading frame (*SPCC584.02*) that encoded an uncharacterized protein which displayed an extended homology at its N terminus to the N terminus of the *S. pombe* copper-sensing transcription factor Cuf1. Because this common region was shared by the two proteins, the locus *SPCC584.02* was named *cuf2^+^*. The N-terminal 60 amino acid segment of Cuf2 displayed 42% sequence identity and 62% sequence similarity with the Cuf1 61 amino acid segment (Fig. 1). A putative zinc coordination domain (residues 1–40) lies within this region of Cuf2 and may be part of a minor groove DNA binding domain that is similar to those found in previously characterized copper metalloregulatory transcription factors [25]. Such regulators are mainly involved in either the copper detoxification or copper acquisition pathways. In particular, the Ace1 and Amt1 proteins from the yeasts *S. cerevisiae* and *C. glabrata*, respectively, promote copper detoxification via the induction of metallothionein gene transcription and possess such a domain [26]. In the yeasts *S. cerevisiae* and *S. pombe*, the Mac1 and Cuf1 proteins, respectively, promote copper acquisition through the induction of the copper transporting genes [20], [27]. Similar to Ace1, Amt1, Mac1 and Cuf1, Cuf2 harbors a conserved (R/K)GRP motif (Fig. 1) that is known to be involved in the direct binding of the nucleotides located within the minor groove of DNA helix [18], [24]. Interestingly, the N-terminal region of Cuf2, specifically residues 11 to 53, contained 9 basic amino acids (Arg^16^, Arg^19^, Arg^28^, Arg^34^, Arg^36^, Arg^38^, Lys^44^, Arg^46^ and Lys^49^) that are highly conserved in Cuf1 and whose presence (underlined amino acids) has been shown to be required for the targeting of Cuf1 to the nucleus [28]. The first N-terminal 60 amino acid residues of Cuf2 also shared a strong sequence homology with the N terminal sequences of the Ace1 and Amt1 proteins. However, Cuf2, as is the case of Cuf1, did not possess the second half of the Ace1/Amt1 copper regulatory domain in which two highly conserved Cys-X-Cys sequence motifs are found [20]. The absence of these two Cys pairs in Cuf2 makes improbable the formation of Ace1/Amt1-like copper regulatory domain that consists of two lobes separated by a cleft in which a Cu_4_S_6_ center takes place in the presence of copper ions [18]. Moreover, as opposed to Cuf1 and Mac1, Cuf2 did not contain a Cys-rich domain (Cys-X-Cys-X_(3/4)_-Cys-X-Cys-X_2_-Cys-X_2_-His) located near its C terminus that could sense and coordinate copper ions [29]–[31]. In fact, the C-terminal region of Cuf2 lacked any potential motif that could bind copper ions. Nevertheless, because some features of Cuf2 were reminiscent of copper-regulatory transcription factors, the *cuf2^+^* gene was isolated for further analysis.

### *cuf2* ^+^ is expressed exclusively during meiosis

**Figure 2:**
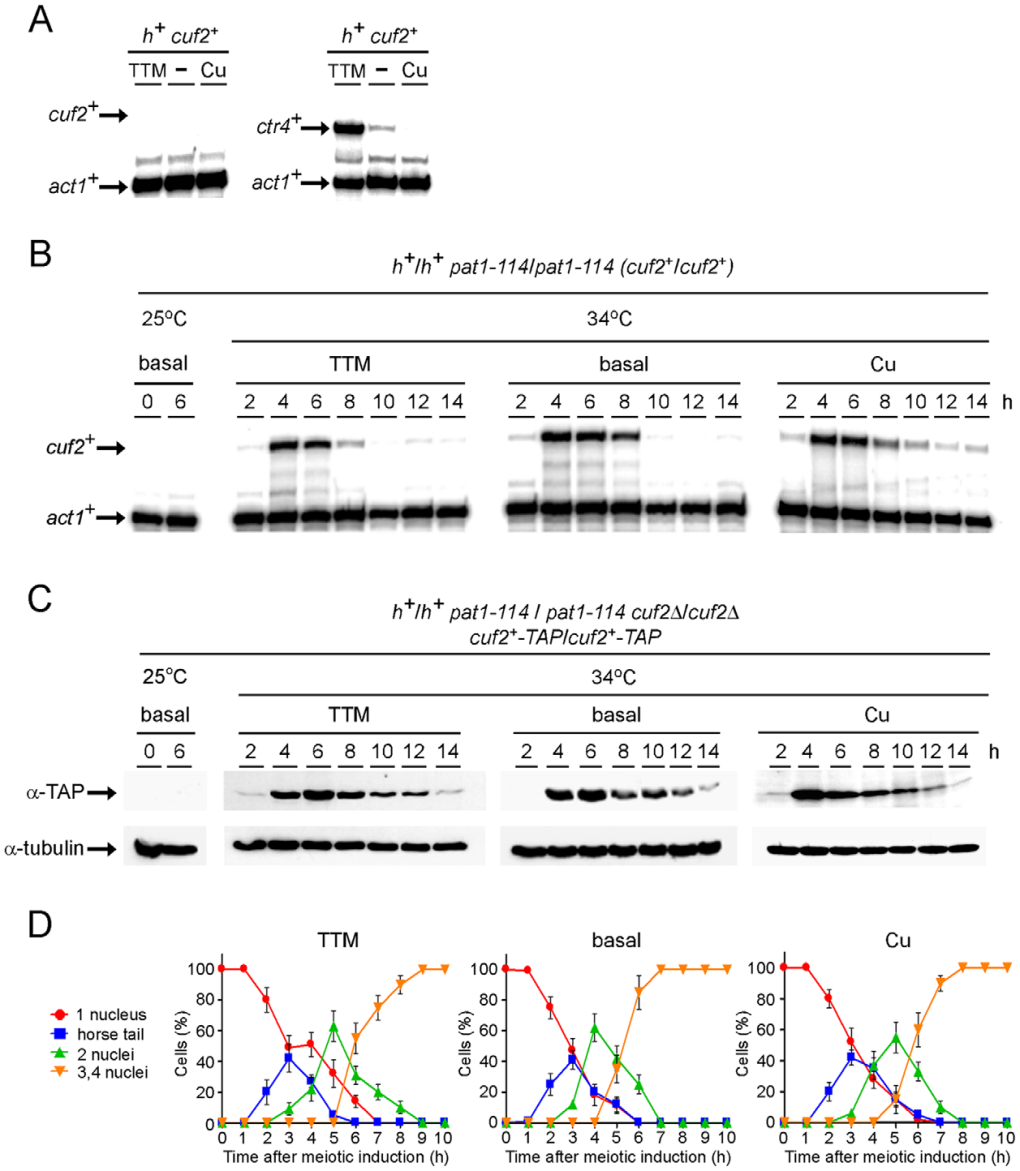
Assessment of the mRNA and protein steady-state levels of Cuf2 during meiosis. *A*, Representative expression profiles of the *cuf2* ^+^ and *ctr4* ^+^ mRNAs in *h* ^+^ haploid cells that were either left untreated (−) or were treated with either 50 µM TTM or 50 µM CuSO_4_ during mitosis. *B*, Cultures of *pat1-114/pat1-114* diploid cells were either maintained in vegetative growth at 25°C, or were induced to initiate and proceed through meiosis at 34°C. *pat1-114/pat1-114* diploid cells were either left untreated, or incubated in the presence of 50 µM TTM or 50 µM CuSO_4_. Total RNA was isolated at the indicated time points after the induction of meiosis. Shown are representative RNase protection assays of both the *cuf2^+^* and the *act1^+^* (internal control) mRNA steady-state levels during meiosis. *C*, Cuf2-TAP protein expression during meiosis. *pat1-114/pat1-114 cuf2Δ/cuf2Δ* diploid cells expressing Cuf2-TAP were either left uninduced (25°C), or were induced (34°C) under basal conditions or in the presence of 50 µM TTM or 50 µM CuSO_4_. Shown are Western blots of both Cuf2-TAP and α-tubulin (control loading) levels at different time points after meiotic induction. *D*, Meiotic progression of cells under either basal (untreated) conditions, or in the presence of TTM (50 µM) or CuSO_4_ (50 µM). The values shown for each condition (TTM, basal or Cu) correspond to the percentage of cells with 1, 2, or 3–4 nuclei, and the percentage of cells with horse tails. The graphed values represent the averages of triplicate determinations +/− the standard deviations.

Initial experiments using cells proliferating in mitosis failed to detect the *cuf2^+^* transcript regardless of the copper status (Fig. 2A). In contrast, as expected, the *ctr4^+^* copper transport mRNA levels (assayed as a control) were up- (∼10-fold) and down- (∼3-fold) regulated after treatment with the copper chelator tetrathiomolybdate (TTM) and copper, respectively, as compared to basal conditions (Fig. 2A). The *cuf2^+^* transcript was first detected in genome-wide studies in which cells were switched from mitosis to meiosis [14]. After cell entrance into meiosis, the expression profile of *cuf2^+^* revealed that it reached a maximum 5 h after meiotic induction, indicating that *cuf2^+^* was a middle meiotic gene potentially involved in the differentiation process [14].

*S. pombe* cells growing mitotically carry an active Pat1 kinase which inhibits cells from entering meiosis. Cells harboring the *pat1-114* mutation show a temperature-sensitive growth and undergo meiosis at the restrictive temperature of 34°C. The use of the *pat1-114* temperature-sensitive mutant permits the synchronization of cells in terms of their entry into the meiotic program [12]. To further investigate the expression profile of *cuf2^+^* during meiosis, a *pat1-114/pat1-114* diploid strain that was pre-synchronized in G1 mitotic-phase by nitrogen starvation at 25°C was used. The temperature was then shifted to 34°C so as to inactivate Pat1 and allow the cells to undergo synchronous meiosis. The results obtained were consistent with data reported by others [14], namely that the expression of *cuf2* ^+^ peaked between 4 and 6 h after meiotic induction (Fig. 2B). A homozygous *pat1-114/pat1-114 cuf2Δ/cuf2Δ* diploid strain was generated to validate the signal corresponding to the *cuf2^+^* RNase protection product (data not shown). In all of the experiments the *cuf2^+^* transcript was found to be absent in the disruption strain (*cuf2Δ/cuf2Δ*), irrespective of the copper status (data not shown). To examine if copper availability had any effect on the expression of *cuf2* ^+^ during the meiotic program, the cells were either left untreated or were treated with either the copper chelator TTM (50 µM) or CuSO_4_ (50 µM) prior to the temperature shift (34°C). Aliquots of cultures were taken every 2 h following meiotic induction, and the steady-state levels of *cuf2^+^* mRNA were analyzed by RNase protection assays. Results showed that the steady-state levels of *cuf2* ^+^ mRNA under basal (untreated), copper-starved (50 µM TTM) or copper replete (50 µM CuSO_4_) conditions were primarily increased between 4 and 6 h following meiotic induction (Fig. 2B). In response to 50 µM copper, the *cuf2^+^* mRNA levels were induced within 4 h, but were down-regulated to a lesser degree over time and were maintained at a level ∼1.4-fold above the basal mRNA levels detected in untreated cells (Fig. 2B). To ascertain whether or not the steady-state levels of Cuf2 protein followed those of the *cuf2^+^* mRNA, a *pat1-114/pat1-114 cuf2Δ/cuf2Δ* strain in which a functional *cuf2^+^-TAP* fusion allele was returned into the genome by integration was used. The results showed that, under basal, low or high copper conditions, Cuf2-TAP protein levels increased in a manner similar to that of the *cuf2^+^* transcript levels, although it should be noted that steady-state levels of Cuf2 protein remained present for a longer period of time than did the mRNA (Fig. 2C). To ensure that cell growth conditions (basal, 50 µM TTM or CuSO_4_) had no negative effect on meiotic progression and sporulation, a series of microscopic analyses were performed. *pat1-114/pat1-114* diploid cells were synchronously induced into meiosis and Hoescht 33342 was added to 0.5 µg/µl every hour to cell culture aliquots to visualize the DNA and monitor meiotic progression. Under basal and copper conditions, meiosis I occurred primarily between 3.5 and 6 h after meiotic induction, meiosis II between 6.5 and 8 h (Fig. 2D) and sporulation after 8 h (data not shown). Although meiotic progression of cells under mild copper starvation conditions (50 µM TTM) was slowed by approximately 1 h as compared to untreated (basal) cells, spore formation was clearly observed at the end of meiosis (data not shown). Globally, cells that were grown under basal, low or elevated (50 µM) copper concentrations displayed no significant changes in their ability to proceed meiosis since there was no apparent timing defect in their progression (Fig. 2D). Taken together, these results indicated that, under basal, copper-depleted and copper-replete conditions, the *cuf2^+^* gene is effectively but transiently expressed during meiosis. Furthermore, the Cuf2 protein was mostly produced during meiotic divisions, with reduced levels of protein that persist towards the end of the meiotic program.

### The *mei4* ^+^ gene is required for *cuf2* ^+^ gene expression

**Figure 3:**
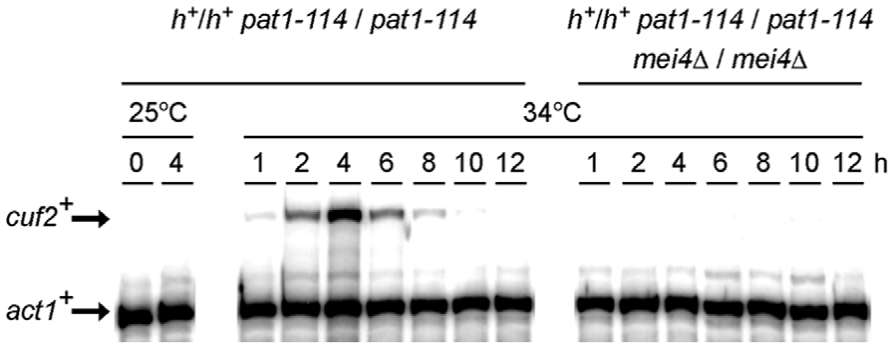
*cuf2* ^+^ gene expression is Mei4-dependent. *pat1-114*/*pat1-114 (mei4^+^/mei4^+^)* and *pat1-114*/*pat1-114 mei4Δ/mei4Δ* strains were pre-synchronized by nitrogen starvation at 25°C, and then were induced to undergo synchronous meiosis at 34°C. At the indicated time points, the *cuf2^+^* and *act1^+^* (internal control) mRNA levels were analyzed in both the control strain (*mei4^+^/mei4^+^*) and the isogenic strain lacking the *mei4^+^* alleles. To validate the absence of the *cuf2^+^* transcript during mitosis, total RNA was probed for the presence of *cuf2^+^* mRNA in vegetative cells incubated at 25°C.

Genome-wide studies of the global effects of the deletions of meiosis-specific transcription factors have revealed that the transcription regulator Mei4 is required for the induction of numerous of the middle meiosis-specific genes [15]. To independently assess whether or not Mei4 was necessary for the expression of the middle meiotic gene *cuf2^+^*, RNase protection assays in synchronous meiosis experiments were carried out using a *pat1-114/pat1-114 mei4Δ/mei4Δ* diploid strain and the results were compared to those obtained with a *pat1-114/pat1-114* control strain. In the case of *pat1-114/pat1-114* control cells, *cuf2^+^* exhibited a typical middle meiosis gene time-dependent expression profile, peaking 4 h after meiotic induction (Fig. 3). In contrast, *cuf2^+^* mRNA was absent in *pat1-114/pat1-114 mei4Δ/mei4Δ* cells throughout meiosis (Fig. 3). Furthermore, the *cuf2^+^* transcript was not detected in the *pat1-114/pat1-114 mei4Δ/mei4Δ* mutant under all of the conditions tested, including basal, copper-replete and copper-depleted conditions (data not shown). The observation that the *pat1-114/pat1-114 mei4Δ/mei4Δ* deletion strain showed a loss of *cuf2^+^* gene expression indicates that the meiotic-dependent expression of *cuf2^+^* mRNA requires Mei4.

### Subcellular localization of Cuf2 during meiosis

**Figure 4:**
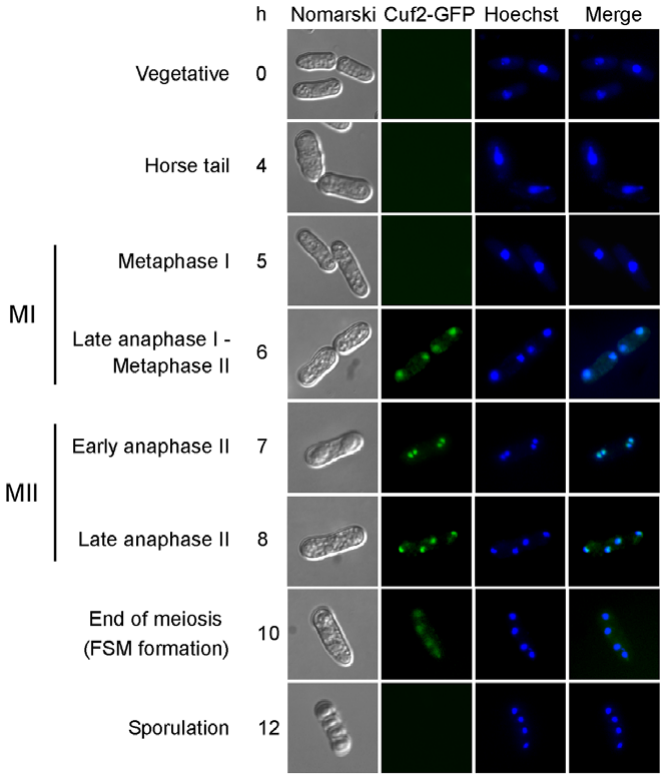
Analysis of Cuf2-GFP localization during both meiosis and sporulation. The Cuf2-GFP fluorescence signal (center left) was observed at different stages of meiosis following the azygotic meiotic induction of a *h^+^/h^−^ cuf2Δ/cuf2Δ cuf2^+^-GFP/cuf2^+^-GFP* strain. Once induced, azygotic meiotic cells were differentiated under basal conditions. Cuf2-GFP was observed at each extremity of the cell at the end of the first meiotic division (MI) that is to say at anaphase I. Cuf2-GFP followed the segregation of chromatids until late in anaphase II when the second meiotic division (MII) occurred in early anaphase II. The Cuf2-GFP fluorescence signal was detected during the FSM formation (i.e. at the end of meiosis), but disappeared during spore formation. Cells at different stages of meiosis were stained using Hoechst 33342 to visualize the DNA (centre right). The merged images are shown in the far right panels. Nomarski optics (far left) were used to monitor cell morphology.

Based on the fact that Cuf2 was a meiosis-specific protein, the next step was the elucidation of its subcellular location during the meiotic program. Hence, a GFP coding sequence was fused in-frame with the 3′-end of the *cuf2^+^* gene. When the Cuf2-GFP fusion protein was expressed in meiosis, it complemented the FSM formation deficiency and triggered the down-regulation of several middle meiotic genes in a manner similar to that of either the wild-type (untagged) or the TAP epitope-tagged Cuf2 (data not shown). In another series of experiments, a functional *cuf2^+^-GFP* allele was integrated into *h^−^ cuf2Δ* and *h^+^ cuf2Δ* cells. After mating, *h^−^/h^+^ cuf2Δ/cuf2Δ cuf2^+^-GFP/cuf2^+^-GFP* diploid cells were cultured so that they undergo azygotic synchronous meiosis. Following the induction of meiosis under basal conditions, the Cuf2-GFP fluorescent protein was first detected in late anaphase I/metaphase II, and co-localized with the homologous chromosomes that had undergone the first meiotic division (Fig. 4). At early anaphase II, meiotic cells displayed Cuf2-GFP fluorescence as two pairs of fluorescent spots that appeared to correspond with the chromosomal material (as marked by Hoechst-staining) that had undergone sister chromatid segregation. At late anaphase II, the Cuf2-GFP fusion protein generated a fluorescent signal that behaved like the chromosomal material, and its fluorescence was redistributed in a manner identical to that of the sister chromatids, generating four distinct fluorescent spots in the zygote (Fig. 4). At the end of anaphase II after sister chromatid segregation, the Cuf2-GFP fluorescent signal progressively decreased (during FSM formation) and finally disappeared after spore formation (Fig. 4). Collectively, microscopic analyses of meiotic cells revealed that a functional Cuf2-GFP localized with the chromosomes that had undergone anaphase I, metaphase II and anaphase II (both early and late). These observations were consistent with a regulatory role for Cuf2 at the DNA level, especially during the end of the first and second meiotic divisions and the early steps of FSM formation.

### A subset of middle-phase meiotic genes show an elevated and sustained expression in cells lacking Cuf2

**Figure 5:**
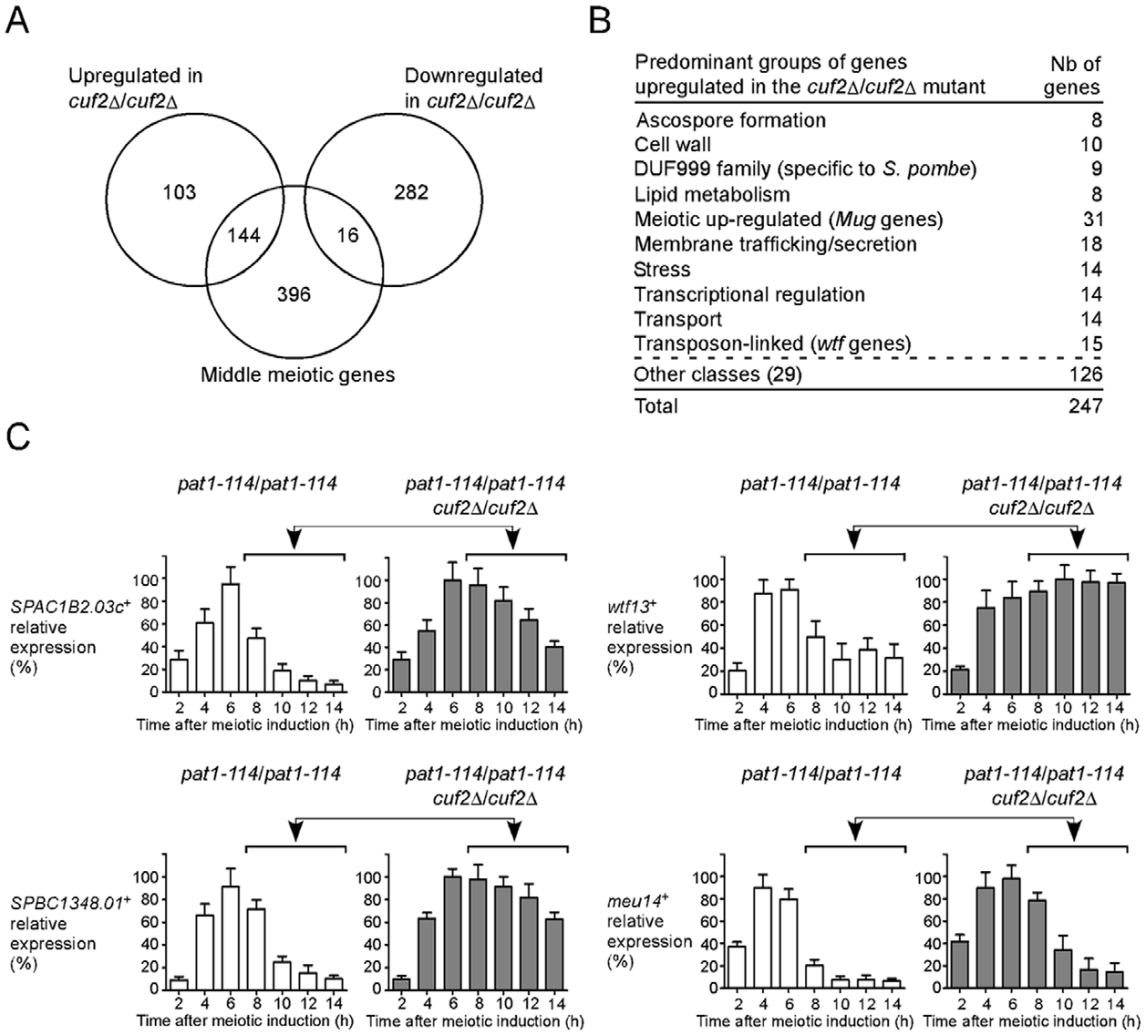
Effect of *cuf2*Δ/*cuf2*Δ deletion on the expression of *S. pombe* genes. *A*, Venn diagram representing the numbers of genes with higher and lower expression levels in the *cuf2Δ/cuf2Δ* disruption strain as compared to the wild-type strain. The diagram also shows the overlap between middle-phase meiotic genes and all differentially expressed genes identified in *cuf2Δ/cuf2Δ* cells. *B*, Genes that exhibit higher expression levels in *cuf2Δ/cuf2Δ* cells were grouped in different functional families. *C*, *pat1-114*/*pat1-114 (cuf2^+^/cuf2^+^)* and *pat1-114*/*pat1-114 cuf2Δ/cuf2Δ* strains were pre-synchronized by nitrogen starvation and were then induced to undergo synchronous meiosis under basal conditions. At the indicated times following meiotic induction, total RNA was prepared from both the *cuf2^+^/cuf2^+^* strain and its *cuf2Δ/cuf2Δ* mutant derivative and was analyzed by RNase protection assay. The histograms shown the quantifications of three independent RNase protection experiments. Ten hours after meiotic induction, the results indicate that the levels of the *SPAC1B2.03c^+^*, *wtf13^+^*, *SPBC1348.01^+^* and *meu14^+^* transcripts in *cuf2Δ/cuf2Δ* mutant cells were derepressed above those observed in *cuf2^+^/cuf2^+^* cells under the same conditions (as indicated with arrows and brackets).

Based on the observations that Cuf2 co-localized with the chromosomal material (Fig. 4) and contained a putative DNA binding region, we hypothesized that Cuf2 could participate in the regulation of the meiotic genes during the sexual differentiation process. This possibility was investigated using a microarray approach to determine whether meiosis-specific genes were differentially expressed as a function of Cuf2 status. A *pat1-114/pat1-114 cuf2Δ/cuf2Δ* mutant strain and a *pat1-114/pat1-114* control strain were pre-synchronized in G1 by nitrogen starvation, and were then incubated at 34°C so as to inactivate the Pat1 kinase. These conditions were set to initiate and proceed through synchronous meiosis under basal conditions. Microarrays were hybridized with probes derived from RNA isolated from either *cuf2Δ/cuf2Δ* mutant cells or control (*cuf2^+^/cuf2^+^*) cells. The analysis of gene expression profiling data obtained nine hours after meiotic induction revealed that 247 genes were expressed at higher levels in the *cuf2Δ/cuf2Δ* mutant cells (averaging ≥1.5-fold) (Fig. 5A; Table S1). Conversely, the data also revealed that 298 genes were expressed at lower levels in the *cuf2Δ/cuf2Δ* mutant cells (averaging ≥1.5-fold) (Fig. 5A). The reason why we selected the 9-h time point for microarray experiments was based on the fact that we wanted to leave time of the translational process and the protein product (Cuf2) to take place and operate after the production of the *cuf2^+^* transcripts in *cuf2^+^*/*cuf2^+^* cells.

As previously reported, the transcription profiles of the meiotic cell cycle have defined four successive waves of gene expression that coincide with the major meiotic phases. These are, wave 1 in response to nutrient starvation (nutrient-responsive genes), wave 2 that involves premeiotic replication and recombination (early meiotic genes), wave 3 during which the meiotic divisions occur (middle meiotic genes), and wave 4 that is associated with spore formation (late meiotic genes) [14]. Among the 247 genes that were expressed at higher levels in the absence of Cuf2, 149 were classified as either early (4 genes), middle (144 genes) or late (1 gene) meiotic genes (Table S2). The other 98 genes were unclassified with respect to the expression waves. Strikingly, 144 out of a total of 149 genes classified were middle meiotic genes (97%). The expression levels of these 144 genes were up-regulated and remained at high steady-state levels even during late meiosis when their expression levels normally were reduced in order to return them to a basal level of expression.

Some of the genes with increased expression levels in the absence of Cuf2 could be grouped together based on their predicted protein products or meiotic-specific profiles of expression. Some groups of proteins displayed conserved homologous domains that were potentially functionally important. One example of these groups included the meiotic genes encoding the family of uncharacterized DUF999 proteins of which 9 members were identified (Fig. 5B; Tables S2, S3 and S4). A second group of 15 genes, which encoded the Wtf family of proteins, was also expressed at higher levels in *cuf2Δ/cuf2Δ* mutant cells. *wtf* genes are known to be flanked by intergenic regions that contained long terminal repeat fragments of retrotransposons, and are transcribed during meiosis [32]. A third group includes several members of the Mug (meiotically up-regulated gene) family of proteins that were abnormally up-regulated in the absence of Cuf2 (Table S4).

Because the results indicated that Cuf2 may have a role in repressing middle meiotic genes, we concentrated our efforts on this aspect in the present study. To assess whether the microarray analyses were successful in identifying the up-regulated genes in *cuf2Δ/cuf2Δ* mutant cells, RNase protection assays were performed using an independent biological trial to examine the relative expression levels of four middle-phase meiotic genes: *SPAC1B2.03c^+^*, *wtf13^+^*, *SPBC1348.01^+^* and *meu14^+^*. In the case of the *cuf2Δ/cuf2Δ* mutant strain, at all points examined after 10 h, the levels of these four mRNAs were up-regulated as compared to those in control *cuf2^+^/cuf2^+^* cells (Fig. 5C). As expected in experiments using either DNA microarray or RNase protection analysis, *SPAC1B2.03c^+^*, *wtf13^+^*, *SPBC1348.01^+^* and *meu14^+^* transcripts exhibited similar increases in transcript abundance in a strain lacking Cuf2. At the 10-h time point, RNase protection experiments revealed that the expression level of *SPAC1B2.03c^+^*, *wtf13^+^*, *SPBC1348.01^+^* and *meu14^+^* were increased 4.4-, 1.6-, 3.6- and 4.5-fold, respectively, while DNA microarray analysis showed increases of 3.1-, 2.4-, 2.5- and 2.2-fold, respectively (Fig. 5C and Table S2).

**Figure 6:**
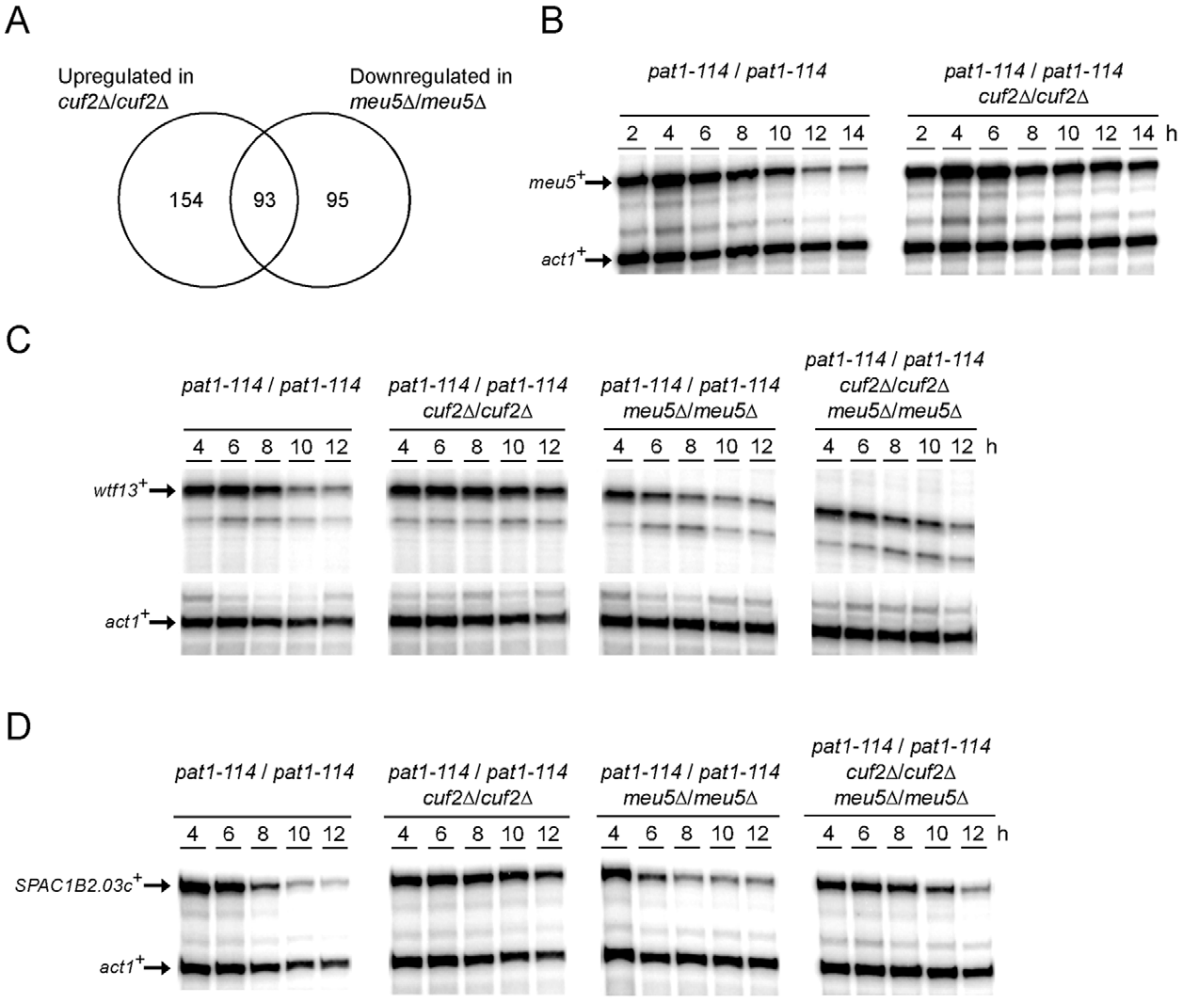
*cuf2*Δ and *meu5*Δ gene disruptions have opposite effects on common meiosis-specific genes. *A*, Venn diagram representing the overlap between the *cuf2^+^*- and the *meu5^+^*-dependent genes. *B*, Total RNA from both the *pat1-114/pat1-114* (*cuf2^+^*/*cuf2^+^*) and *pat1-114*/*pat1-114 cuf2Δ/cuf2Δ* disruption strains was analyzed throughout meiosis and sporulation. A representative RNase protection experiment of the effect of the absence of Cuf2 on the expression of the *meu5^+^* transcript (especially 10 to 14 h after meiotic induction) is shown. *C–D*, Cultures of *pat1-114/pat1-114*, *pat1-114*/*pat1-114 cuf2Δ/cuf2Δ*, *pat1-114*/*pat1-114 meu5Δ/meu5Δ* and *pat1-114*/*pat1-114 cuf2Δ/cuf2Δ meu5Δ/meu5Δ* diploid cells were synchronously induced into meiosis under basal conditions. Total RNA was isolated from culture aliquots taken at the indicated time points. After RNA preparation, the *wtf13^+^* and *SPAC1B2.03c^+^* steady-state mRNA levels were analyzed by RNase protection assays using actin (*act1* ^+^) as an internal control. The results shown are representative of three independent experiments.

Interestingly, *cuf2Δ/cuf2Δ* cells showed sustained higher levels of *meu5* ^+^ expression (2.3-fold up-regulated according to the DNA microarray analysis) as compared with those observed in the control (*cuf2^+^/cuf2^+^*) cells. The *meu5* ^+^ gene encodes a RNA-binding protein that stabilizes meiosis-specific transcripts, especially those expressed in the middle meiotic phase [33]. To further investigate the relation between the *cuf2^+^* and *meu5* ^+^ genes, the *meu5* ^+^ regulon [33] was compared to that of *cuf2^+^*. In all 93 genes were found to be common to both regulons (Fig. 6A; Table S3). Based on this observation, we hypothesized that, in the absence of Cuf2, *meu5* ^+^ expression was up-regulated. As a consequence, the Meu5 protein was present for a longer period of time, thereby stabilizing and extending the presence of several middle-phase meiotic transcripts to the later time points. To independently verify the microarray data, the diploid strains *pat1-114 cuf2^+^/cuf2^+^* and *pat1-114 cuf2Δ/cuf2Δ* were synchronized through meiosis and the *meu5* ^+^ mRNA levels were monitored at different time points after meiotic induction. Consistent with the microarray results, the *meu5* ^+^ mRNA levels were ∼2.4-fold higher (in *cuf2Δ/cuf2Δ* cells as compared to those in the *cuf2^+^/cuf2^+^* control cells) after 10 h of meiotic induction and remained elevated (∼4.2-fold higher) even after both 12 and 14 h of meiotic induction (Fig. 6B). To further investigate the effect of Meu5 on the expression profile of Cuf2-regulated target genes, two additional diploid mutant strains, *pat1-114 meu5Δ/meu5Δ* and *pat1-114 cuf2Δ/cuf2Δ meu5Δ/meu5Δ*, were created. In the absence of Cuf2 (*cuf2Δ/cuf2Δ*), *wtf13* ^+^ and *SPAC1B2.03^+^* transcripts were found to be significantly increased after 10 h of meiotic induction (Figs. 5C, 6C and D). In contrast, only very low amounts of the *wtf13* ^+^ and *SPAC1B2.03^+^* transcripts were detected in *pat1-114 meu5Δ/meu5Δ* cells (Fig. 6, C and D). These results correlate well with the concept that Meu5 stabilizes and extends the presence of *wtf13* ^+^ and *SPAC1B2.03^+^* mRNAs to the later meiotic time points. In cells lacking both Cuf2 and Meu5 (*cuf2Δ/cuf2Δ meu5Δ/meu5Δ*), the levels of *wtf13* ^+^ and *SPAC1B2.03^+^* mRNAs were found to be slightly higher as compared with those observed in *pat1-114 meu5Δ/meu5Δ*, but lower as compared with those detected in *pat1-114 cuf2Δ/cuf2Δ* cells (Fig. 6, C and D). Taken together, the results strongly suggest that Cuf2 is a meiosis-specific regulator that functions in a timely, controlled repression of middle genes during meiotic differentiation. Furthermore, the results suggest that Cuf2 and Meu5 have opposite effects on common meiosis-specific genes, specifically that Cuf2 down-regulates middle-phase transcripts, while Meu5 stabilizes the same transcripts.

### Deletion of *cuf2* ^+^ leads to FSM defects

**Figure 7:**
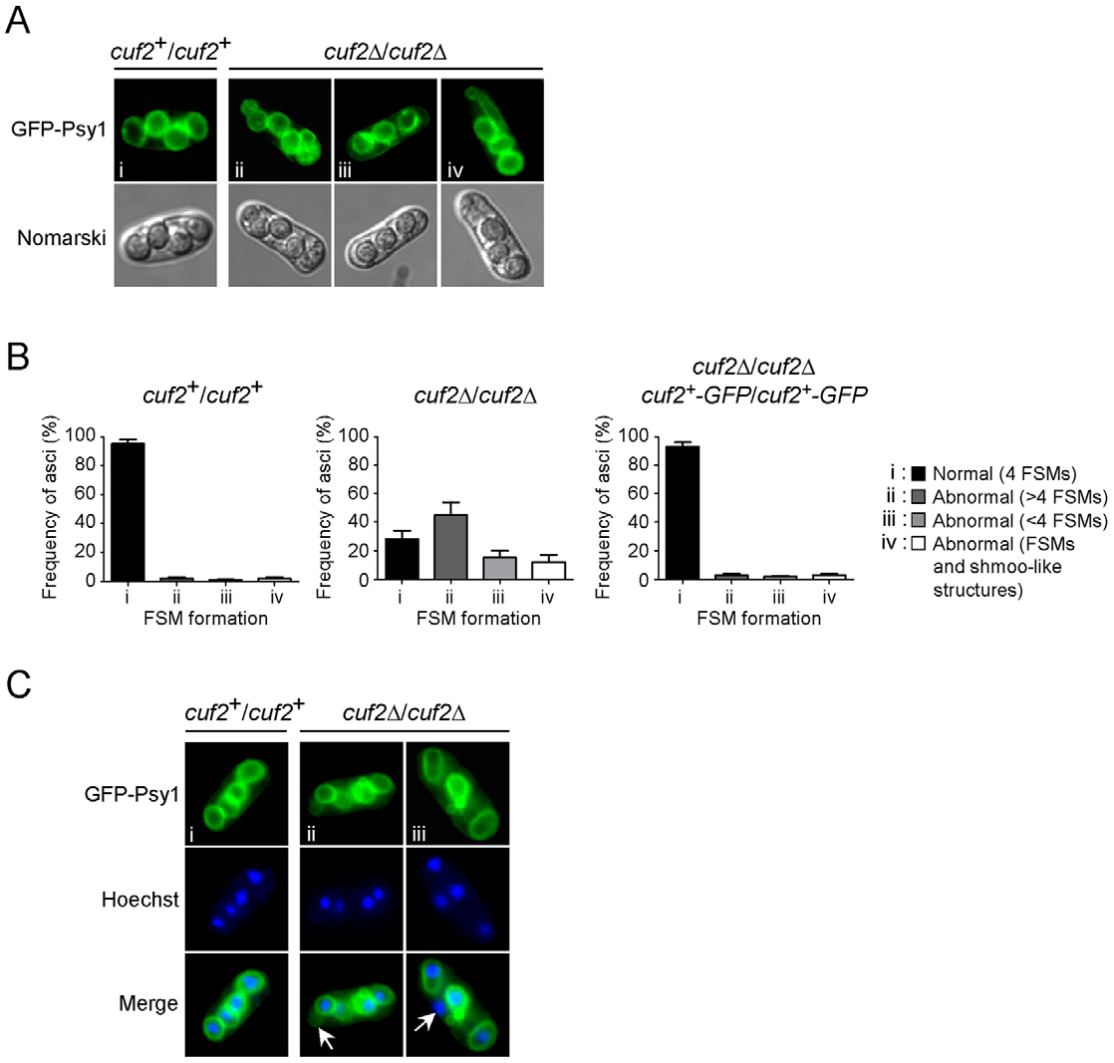
The *cuf2*Δ/*cuf2*Δ mutant is defective in FSM formation. *A*, Both wild-type diploid (*cuf2^+^/cuf2^+^*) and *cuf2Δ/cuf2Δ* mutant cells expressing GFP-Psy1 were synchronously induced to undergo azygotic meiosis. At the 9 h time point, FSM formation was monitored by detecting the GFP-Psy1 signal by fluorescence microscopy. When GFP-Psy1 localized to 4 circular FSM structures, these FSMs were classified as normal (i). When GFP-Psy1 localized to either lesser or greater than 4 circular FSMs or shmoo-like structures, FSMs were classified as abnormal (ii, iii and iv). *B*, Histograms showing the percentages of each normal (i) and abnormal (ii, iii, iv) FSM structure in both wild-type (*cuf2^+^/cuf2^+^*) and *cuf2Δ/cuf2Δ* mutant cells, as well as in a diploid *cuf2Δ/cuf2Δ* disruption strain in which wild-type copies of the *cuf2^+^-GFP* fusion gene were reintegrated. *C*, Typical images of FSM structures 9 h after meiotic induction in both wild-type (*cuf2^+^/cuf2^+^*) and *cuf2Δ/cuf2Δ* mutant cells (top panels). Each strain had previously been transformed with pJK210GFP-Psy1, which encodes GFP-Psy1 that is used as an FSM-resident marker. Hoechst 33342 staining was used to visualize the chromosomal DNA (middle panels). The merged images of the GFP-Psy1 and the Hoechst 33342 dye are shown in the bottom panels. Anucleated FSM structures, or unpackaged nuclei, are indicated by the white arrows.

The results shown above revealed that the expression levels of several middle-phase meiotic genes were sustained, even during late meiotic time points in the absence of Cuf2 (Fig. 5 and Table S2). One possible explanation for this is that Cuf2 could be specifically required for the down-regulation processes that normally occurred only during the divisions steps. The absence of Cuf2 would trigger errors that could jeopardize both the quality and quantity of forespores. To test this possibility the meiotic differentiation in *h* ^+^/*h* ^−^ *cuf2Δ/cuf2Δ* diploid cells was compared to that of *h* ^+^/*h* ^−^ *cuf2^+^/cuf2^+^* control cells (Fig. 7A). Both strains were pre-synchronized in G1 by nitrogen starvation and were then synchronously induced to undergo azygotic meiosis. In the case of the control cells, 4 FSMs were detected 8 to 9 h after meiotic induction (Fig. 7A). The presence of FSMs was confirmed using a GFP-tagged Psy1 protein as Psy1 is a well-established FSM marker [34]. An abnormal number of FSMs were observed in the case of the *cuf2Δ/cuf2Δ* mutant cells. Specifically, ∼42% of the mutant cells exhibited more than 4 FSMs per ascus (Fig. 7, A and B, group ii), whereas ∼18% of cells displayed 3 FSMs per ascus (Fig. 7, A and B, group iii). Many *cuf2Δ/cuf2Δ* cells showed FSMs of various sizes (∼15% of cells), including elongated FSMs with small buds forming a shape reminiscent of a shmoo (Fig. 7, A and B, group iv) as previously reported [35]. To confirm that the meiotic FSM maturation defect was due to the inactivation of *cuf2^+^*, a *h* ^+^/*h* ^−^ *cuf2Δ/cuf2Δ* strain was created in which wild-type *cuf2^+^-GFP/cuf2^+^-GFP* alleles were returned by integration and expressed under the control of the *cuf2^+^* promoter. In these experiments, normal FSM formation was observed when the strain underwent azygotic meiosis in a manner similar to that observed in control cells (Fig. 7B).

Further insight into the phenotype resulting from loss of Cuf2 function was gained by determining the presence of chromosomal DNA within each FSM structure. All asci generated from *cuf2^+^/cuf2^+^* cells contained chromosomal DNA (Hoescht 33342-staining) and was surrounded by FSMs (Fig. 7C). Although 4 chromosomal DNA spots were also found in *cuf2Δ/cuf2Δ* mutant cells, the chromosomal DNA spots were not all packaged into FSM structures (Fig. 7C and data not shown). Taken together, these results suggest that loss of Cuf2 leads to the formation of asci containing unpackaged chromosomal DNA in addition to the anucleated FSM shells observed with the shmoo-like FSM structures.

### Spore viability is reduced in *cuf2Δ/cuf2Δ* mutant cells

**Figure 8:**
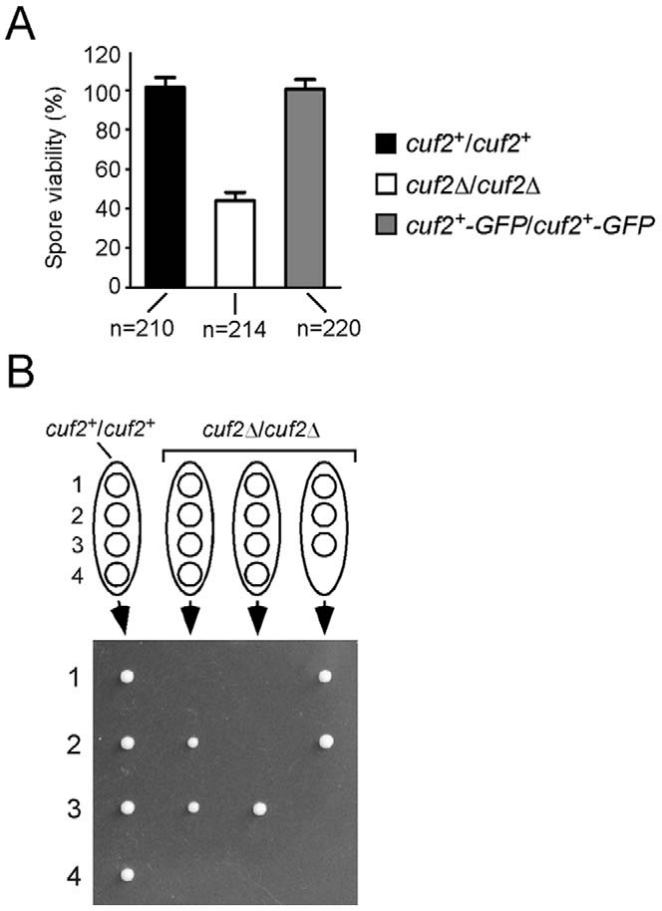
*cuf2*Δ/*cuf2*Δ mutant cells display reduced spore viability. *A*, Both wild-type (*cuf2^+^/cuf2^+^*) and *cuf2Δ/cuf2Δ* mutant cells were allowed to sporulate under conditions of limiting nitrogen. Tetrad dissection analysis (>200 asci per strain; n = 210 and 214, respectively) revealed that ∼59% of the spores from *cuf2Δ/cuf2Δ* asci failed to germinate as compared to spores from wild-type (*cuf2+/cuf2+*) asci (∼98% germination). As an additional control, the mutant strain bearing the disrupted *cuf2Δ/cuf2Δ* alleles was used to reintegrate functional *cuf2^+^-GFP/cuf2^+^-GFP* alleles and was then examined for the formation of 4-spored asci. This control strain exhibited 97% of 4-spore asci (n = 220). *B*, Comparative typical results of tetrad dissection analysis in wild-type (*cuf2^+^/cuf2^+^*) and *cuf2Δ/cuf2Δ* mutant asci.

We next asked whether the inactivation of Cuf2 that caused abnormal FSM structures was intrinsically linked to a decrease of spore viability. To answer this question *h* ^+^/*h* ^−^ *cuf2Δ/cuf2Δ* diploid cells were used, and the results obtained compared to those from either *h* ^+^/*h* ^−^ *cuf2^+^/cuf2^+^* or *h* ^+^/*h* ^−^ *cuf2Δ/cuf2Δ* cells in which a functional integrative plasmid harboring the *cuf2^+^-GFP* allele expressed under the control of the *cuf2^+^* promoter was present. After 12 h of induced azygotic meiosis, the cells were analyzed by performing tetrad dissection assays. Spores dissected from *h* ^+^/*h* ^−^ *cuf2Δ/cuf2Δ* asci exhibited a ∼59% decrease in viability as compared those of both wild-type and *h* ^+^/*h* ^−^ *cuf2Δ/cuf2Δ cuf2^+^-GFP/cuf2^+^-GFP* cells (Fig. 8A).

Tetrad dissections were also performed to determine whether *h* ^+^/*h* ^−^ *cuf2Δ/cuf2Δ* asci containing 4 spores were as viable as those containing 3 spores (Fig. 8B). Spores originating from asci containing 3 or 4 spores had reduced viabilities when returned to rich medium for germination. *h* ^+^/*h* ^−^ *cuf2Δ/cuf2Δ* asci containing 3 and 4 spores showed ∼58% and ∼42% decreases in viability, respectively, as compared to spores from control strains (data not shown). As shown in Fig. 8B, various viability ratios were obtained after the germination of spores originating from *h* ^+^/*h* ^−^ *cuf2Δ/cuf2Δ* cells (4/4 (29%), 3/4 (19%), 2/4 (29%), 1/4 (14%) and 0/4 (9%); data not shown). Taken together, the results revealed that Cuf2 is important during the meiotic differentiation program in the proper formation of the FSM, which in turn is a prerequisite for spore viability.

## Discussion

Blast searches of the *S. pombe* proteome identified two proteins that contain a putative copper-fist-like domain. The first protein, Cuf1, activates the *ctr4* ^+^, *ctr5* ^+^ and *ctr6* ^+^ genes, which all encode copper transport proteins [20], [21], [23], [31], [36]–[38]. The second protein containing a putative copper-fist-like domain was Cuf2 for which relatively little data was available [14]. Curiously, Cuf2 was found to be strictly expressed during meiosis. Because on this, Cuf2 became highly interesting as it represented the first example of a meiosis-specific copper-fist-like transcription factor. Consequently, the *cuf2^+^* gene was isolated and analyzed. Transcriptome analyses of *S. pombe* cells undergoing synchronized meiosis permitted the identification of meiosis-specific transcripts. In these analyses, *cuf2* ^+^ was consistently identified as being a meiosis-specific middle gene, exhibiting a peak of expression between 4 and 6 h after the induction of meiosis. Middle meiotic genes are transiently expressed during meiotic divisions when the segregation of the homologous chromosomes (MI), and ultimately of sister chromatids (MII) occurs. The meiosis-specific forkhead-type transcription factor Mei4 activates the expression of the majority of the middle meiotic genes [15], [17]. Previous microarray experiments have suggested that *cuf2* ^+^ expression requires Mei4 [15]. In agreement, the results presented here demonstrate that *cuf2* ^+^ transcription relies on Mei4, since *cuf2* ^+^ expression was completely abolished in a *mei4*Δ/*mei4*Δ mutant strain (Fig. 3). Analysis of the *cuf2* ^+^ promoter revealed that it contains four putative consensus Mei4-binding FLEX sequences (positions −49 to −55, −335 to −341, −1104 to −1110 and −1209 to −1215) (the nucleotide numbers refer to the position relative to the A of the initiator codon of the *cuf2^+^* gene) (data not shown). This observation is consistent with the genetic data and further suggests that Mei4 could directly activate *cuf2^+^* expression.

The transcription of *cuf2* ^+^ was unaffected when meiotic cells were treated with ≤50 µM TTM or ≤50 µM CuSO_4_. However, cells that had been treated with ≥75 µM CuSO_4_ consistently exhibited a timing defect in their meiotic progression (data not shown). Under toxic copper concentrations (e.g. ≥75 and 100 µM), *mei4* ^+^ and *cuf2^+^* expression profiles were delayed and overall reduced during the meiotic process (data not shown). These results suggested that toxic levels of copper could negatively affect the expression of several middle meiotic genes. Furthermore, we consistently observed that toxic copper levels (≥75 and 100 µM) affected the ability of meiotically induced cells to complete meiosis. Under these toxic levels of copper (≥75 and 100 µM), the ascospore maturation was abnormal (data not shown). These observations suggested that an excess of copper has a generic negative effect on meiotic gene expression, progression and maturation, rather than specifically affecting the expression of *cuf2* ^+^ and/or *mei4* ^+^ (data not shown). This effect may be explained by the fact that in excess (≥75 and 100 µM), copper may induce intracellular oxidative stress through the generation of hydroxyl radicals produced by the Fenton reaction [39]. Nevertheless, considering its copper-fist-like structure, it is not impossible that Cuf2 may be affected by the cellular status of copper.

The results presented indicate that the expression of *SPAC1B2.03c^+^*, *SPBC1348.01^+^*, *wtf13* ^+^ and *meu14* ^+^ middle genes is up-regulated in a *cuf2*Δ/*cuf2*Δ mutant strain ∼10 to 14 h after meiotic induction. Moreover, analysis of genome-wide gene expression 9 h after meiotic induction revealed that the expression of ∼247 genes was up-regulated (averaging ≥1.5-fold) in a *cuf2*Δ/*cuf2*Δ mutant strain. Interestingly, these genes were associated with a large spectrum of possible cellular functions (Fig. 5B and Table S4), indicating that Cuf2 could act as a middle phase meiotic regulator rather than as a regulator of the gene expression of a specific homeostatic pathway. The microarray results also showed that 298 genes were down-regulated in *cuf2*Δ/*cuf2*Δ mutant cells (Table S5). Because several of these genes encode ribosomal proteins (∼67), which may reflect a global decrease of the cellular metabolic status, we first concentrated our efforts on the analysis of the genes that were up-regulated in the absence of Cuf2.

In all conditions, *cuf2^+^* mRNA was primarily detected between 4 to 6 h after meiotic induction. At the protein level, Cuf2 was first detected at the 4-h time point and was then maintained up to ∼12 h following induction of meiosis. Cuf2-GFP was consistently observed when the first meiotic division occurred ∼5–6 h after meiotic induction. The Cuf2-GFP fluorescent signal progressively decreased 10 to 12 h after meiotic induction, coinciding with mature spore appearance. Interestingly, Cuf2 repression activity was only observed ∼9–10 h following the induction of meiosis, coinciding with the end of the meiotic divisions and the process of spore formation. Although the possibility that Cuf2 could have other functions or target genes between 4 and 8 h following induction of meiosis cannot be excluded, the results suggest that Cuf2 activity is regulated through middle meiosis. This suggestion is based on the observation that Cuf2 became active only once the second meiotic division was completed. It is possible that Cuf2 is subjected to post-translational modifications, or that it interacts with potential co-repressors that are induced downstream of itself.

In the baker's yeast *S. cerevisiae*, the transcriptional regulator Sum1 prevents the expression of meiosis-specific genes in cells undergoing vegetative growth (i.e. in the mitotic cell cycle) [40]. Furthermore, it has been shown that Sum1 represses the expression of a subset of the middle meiotic genes during both early meiosis [41] and at the beginning of late meiosis [42]. Interestingly, the data presented here concerning the repression of middle meiotic genes by Cuf2 was reminiscent of the results obtained with the *S. cerevisiae* protein Sum1. However, Cuf2 is likely to be different from Sum1 as the *SUM1* gene is expressed during mitosis, whereas *cuf2^+^* is not. During meiotic differentiation, *SUM1* is expressed in both the early- and the late-phases of meiosis, as well as during spore maturation, but not in the middle-phase [42], [43]. In contrast, Cuf2 is expressed both in the middle-phase of meiosis and at the onset of late meiosis. In *S. cerevisiae*, it has been shown that the expression of Ndt80, the ortholog of *S. pombe* Mei4, is up-regulated in a *sum1*Δ null strain [42]. In contrast, in *S. pombe*, the *mei4* ^+^ transcript was expressed at comparable levels in both the *cuf2*Δ/*cuf2*Δ mutant and the wild-type parental strains (data not shown). An alignment of the amino acid sequence of Cuf2 with that of Sum1 revealed no sequence similarities between the two proteins (data not shown). Collectively, all of these observations suggest that the Cuf2 and Sum1 functions diverge.

The *S. pombe meu5* ^+^ gene is a meiosis-specific middle gene that encodes an RNA-binding protein which stabilizes transcripts from numerous genes that are expressed throughout middle-phase meiosis [33]. This post-transcriptional regulatory mechanism enhances the translation of ∼188 transcripts, including that of *wtf13* ^+^ [33]. In contrast, Cuf2 repressed the expression of several middle-phase genes at the conclusion of the meiotic divisions, thereby presumably reducing their translation rates during spore maturation (late-phase). Thus, Cuf2 and Meu5 have antagonistic roles with respect to the expression of middle-phase meiotic genes. Interestingly, a significant proportion (93/188) of the Meu5-stabilized transcripts was observed to be expressed at higher levels in *cuf2*Δ/*cuf2*Δ mutant cells than in wild-type cells. Moreover, the *meu5* ^+^ gene itself was expressed at higher levels in a *cuf2*Δ/*cuf2*Δ mutant than in a wild-type strain, especially 10 to 14 h after meiotic induction. This suggests that Cuf2 represses not only *meu5* ^+^ expression, but also that of approximately half of the genes encoding Meu5-stabilized transcripts. This synergic gene regulatory mechanism may optimize the extinction of middle-phase meiotic gene expression at the onset of late meiosis.

The characterization of the molecular mechanisms underlying meiosis revealed that the loss of key meiotic genes may lead to severe defects and could impair spore viability. In the absence of Cuf2, FSM formation and ascospore maturation were abnormal. Considering the fact that during a given meiotic phase, global gene expression of the previous phase is down-regulated [14], [15], the results suggest that the aberrant expression of the middle meiotic genes during late meiosis could have impaired both FSM formation and ascospore maturation. Furthermore, tetrad dissection experiments revealed that spore viability was reduced in a *cuf2*Δ/*cuf2*Δ mutant strain. Surprisingly, 4 nuclei were systematically detected at the end of meiosis in *cuf2*Δ/*cuf2*Δ mutant asci, suggesting that the meiotic divisions were normal. However, a fraction of asci produced by *cuf2*Δ/*cuf2*Δ mutants revealed some anucleated FSMs. These results suggested that FSM defects could have reduced the spore viability in *cuf2*Δ/*cuf2*Δ mutant cells. Taken together, these results further suggested that the Cuf2-dependent timely repression of the middle meiotic genes is critical to providing the correct gene product at the correct time, thereby contributing to normal FSM biogenesis and optimal spore viability.

## Materials and Methods

### Yeast strains and media

The *S. pombe* strains used in this study are listed in Table 1. Standard methods were used for the growth, mating and sporulation of fission yeast cells [44]. Untransformed strains were maintained on yeast extract plus supplements (YES) containing 225 mg/L of adenine, histidine, leucine, uracil and lysine. The strains for which plasmid integration was required were grown on synthetic Edinburgh minimal medium (EMM) lacking the specific nutrients required for selection and maintenance of the recombinant plasmid. The *h^+^*/*h^−^* diploid strains used for azygotic meiosis were isolated as follows. Haploid cells of the opposite mating types were fused on a solid malt extract (ME) medium and the resulting zygotes were then returned to rich media (YES) prior to commitment to meiosis. At this point, the zygotes can resume vegetative growth as diploid cells and later on undergo azygotic meiosis upon a nitrogen (N)-starvation shock. Azygotic meiosis was induced using EMM lacking nitrogen (EMM-N) and supplemented with 10 mg/L of the required auxotrophic nutrients. Diploid strains homozygous for the mating type (*h^+^*/*h^+^*) were generated by protoplast fusion as described previously [45].

**Table 1:** *S pombe* strains used in this study.

Strain	Genotype	Source
FY435	*h^+^ his7-366 leu1-32 ura4-Δ18 ade6-M210*	[48]
FY436	*h^−^ his7-366 leu1-32 ura4-Δ18 ade6-M216*	[48]
FBY13	*h^+^ his3-D1 leu1-32 ura4-Δ18 ade6-M210*	This study
FBY14	*h^−^ his3-D1 leu1-32 ura4-Δ18 ade6-M216*	This study
RAY1	*h^+^ his7-366 leu1-32 ura4-Δ18 ade6-M210 cuf2Δ::KAN^r^*	This study
RAY2	*h^−^ his7-366 leu1-32 ura4-Δ18 ade6-M216 cuf2Δ::KAN^r^*	This study
RAY3	*h^+^ his3-D1 leu1-32 ura4-Δ18 ade6-M210 cuf2Δ::KAN^r^*	This study
RAY4	*h^−^ his3-D1 leu1-32 ura4-Δ18 ade6-M216 cuf2Δ::KAN^r^*	This study
RAY5	*h^+^ his7-366 leu1-32 ura4-Δ18 ade6-M210 cuf2Δ::KAN^r^ cuf2^+^-GFP*	This study
RAY6	*h^−^ his7-366 leu1-32 ura4-Δ18 ade6-M216 cuf2Δ::KAN^r^ cuf2^+^-GFP*	This study
RAY7	*h^+^/h^−^ his7-366/his7-366 leu1-32/leu1-32 ura4-Δ18/ura4-Δ18 ade6-M210/ade6-M216*	This study
RAY8	*h^+^/h* ^−^ *his3-D1/his3-D1 leu1-32/leu1-32 ura4-Δ18/ura4-Δ18 ade6-M210/ade6-M216*	This study
RAY9	*h^+^/h^−^ his7-366/his7-366 leu1-32/leu1-33 ura4-Δ18/ura4-Δ18 ade6-M210/ade6-M216*	This study
	*cuf2Δ::KANr/cuf2Δ::KANr*	
RAY10	*h^+^/h^−^ his3-D1/his3-D1 leu1-32/leu1-33 ura4-Δ18/ura4-Δ18 ade6-M210/ade6-M216*	This study
	*cuf2Δ::KANr/cuf2Δ::KANr*	
RAY11	*h^+^/h^−^ his7-366/his7-366 leu1-32/leu1-33 ura4-Δ18/ura4-Δ18 ade6-M210/ade6-M216*	This study
	*cuf2Δ::KANr/cuf2Δ::KANr cuf2^+^-GFP/cuf2^+^-GFP*	
JB484	*h^+^ pat1-114 ade6-M210*	[11]
JB485	*h^+^ pat1-114 ade6-M216*	[11]
RAY12	*h^+^ pat1-114 ade6-M210 cuf2Δ::KAN^r^*	This study
RAY13	*h^+^ pat1-114 ade6-M216 cuf2Δ::KAN^r^*	This study
RAY14	*h^+^ pat1-114 ade6-M210 mei4Δ::KAN^r^*	This study
RAY15	*h^+^ pat1-114 ade6-M216 mei4Δ::KAN^r^*	This study
RAY16	*h^+^ pat1-114 ade6-M210 cuf2Δ::KAN^r^ cuf2^+^*	This study
RAY17	*h^+^ pat1-114 ade6-M210 cuf2Δ::KAN^r^ cuf2^+^-TAP*	This study
RAY18	*h^+^/h^+^ pat1-114/pat1-114 ade6-M210/ade6-M216*	This study
RAY19	*h^+^/h^+^ pat1-114/pat1-114 ade6-M210/ade6-M216 cuf2Δ::KAN^r^/cuf2Δ::KAN^r^*	This study
RAY20	*h^+^/h^+^ pat1-114/pat1-114 ade6-M210/ade6-M216 mei4Δ::KAN^r^/mei4Δ::KAN^r^*	This study
RAY21	*h^+^/h^+^ pat1-114/pat1-114 ade6-M210/ade6-M216 cuf2Δ::KAN^r^/cuf2Δ::KAN^r^*	This study
	*cuf2^+^/cuf2^+^*	
RAY22	*h^+^/h^+^ pat1-114/pat1-114 ade6-M210/ade6-M216 cuf2Δ::KAN^r^/cuf2Δ::KAN^r^*	This study
	*cuf2^+^-TAP/cuf2^+^-TAP*	
RAY23	*h^+^ pat1-114 ade6-M210 meu5Δ::KAN^r^*	This study
RAY24	*h^+^ pat1-114 ade6-M216 meu5Δ::KAN^r^*	This study
RAY25	*h^+^/h^+^ pat1-114/pat1-114 ade6-M210/ade6-M216 meu5Δ::KAN^r^/meu5Δ::KAN^r^*	This study
RAY26	*h^+^ pat1-114 ade6-M210 cuf2Δ::loxP meu5Δ::KAN^r^*	This study
RAY27	*h^+^ pat1-114 ade6-M216 cuf2Δ::loxP meu5Δ::KAN^r^*	This study
RAY28	*h^+^/h^+^ pat1-114/pat1-114 ade6-M210/ade6-M216 cuf2Δ::loxP/cuf2Δ::loxP*	This study
	*meu5Δ::KANr/meu5Δ::KANr*	

### *pat1*-induced meiosis

In order to synchronize *pat1-114*/*pat1-114* diploid cells for their entry into the meiotic program, the cells were pre-cultured in EMM supplemented with adenine (225 mg/L) at 25°C. The cells were harvested at mid-log phase (1×10^7^ cells/mL) and washed twice before being transferred to EMM-N supplemented with 10 mg/L of adenine. After incubating for 16 h at 25°C, 0.5 mg/mL of NH_4_Cl was added to the culture medium and the culture divided into three. The three fractions were either left untreated, or were treated with either 50 µM of TTM (323446; Sigma-Aldrich) or CuSO_4_ concentrations that ranged from 25 to 100 µM. At this point, the temperature was shifted to 34°C so as to induce meiosis. Meiosis progression was monitored by adding Hoechst 33342 stain to 5 µg/mL (Invitrogen) at different times following meiotic induction.

### Plasmids

As shown in the Results section, the *cuf2* ^+^ gene was found to be exclusively expressed during meiosis. Furthermore, as reported by others [46], it was determined that the gene contained two introns located within its 5′-end's coding region (data not shown). Because of these features, a synthetic DNA fragment possessing the first 52 codons of *cuf2* ^+^ that was optimized for translation in both *S. pombe* and *E. coli* was fused in-frame to the genomic *cuf2* ^+^ codons 53 through 177. To generate the synthetic intron-less DNA segment of *cuf2* ^+^ (i. e. codons 1–52), four oligonucleotides that were partially complementary to each other were annealed in a pairwise manner, forming a partially double-stranded DNA molecule. This molecule was then made completely double-stranded by incubating it with the Klenow fragment of DNA polymerase I in the presence of the four deoxynucleotide triphosphates. The resulting synthetic DNA fragment was digested with PstI and AflII, as these restriction sites had previously been inserted on either side of the desired fragment, and the fragment was then purified using a EZ-10 spin column (Bio Basic, Markham, ON). Polymerase chain reaction (PCR) amplification of the 3′-end DNA segment of *cuf2* ^+^ (i. e. codons 53–177, excluding the stop codon) was carried out using primers designed to generate AflII and SmaI restriction sites at the upstream and downstream termini of the segment, respectively. Genomic DNA from the FY435 *S. pombe* wild-type strain was used as DNA template in this step as no introns have been mapped within this region. The PstI-AflII synthetic intron-less DNA fragment (codons 1–52) and the AflII-SmaI PCR-amplified DNA fragment (codons 53–177) were then ligated together in the PstI-SmaI sites of the pBluescript SK vector (Stratagene, La Jolla, CA), creating pSK*cuf2* ^+^. To create a plasmid possessing the *cuf2* ^+^ gene in-frame with the TAP tag, the coding sequence of TAP was PCR amplified from pJK-148*TAP-fep1^+^* [47]. The resulting DNA fragment was digested with SmaI and SacI, and then fused in-frame with *cuf2* ^+^ into the corresponding sites of pSK*cuf2* ^+^, generating pSK*cuf2* ^+^-*TAP*. To generate the pSK*cuf2* ^+^-*GFP* plasmid, a SmaI-SacI PCR-amplified DNA segment containing the GFP coding sequence was isolated from the plasmid pBP*ctr4* ^+^-*GFP* [22], and was then substituted for the SmaI-SacI restriction fragment present in the plasmid pSK*cuf2* ^+^-*TAP*, thereby replacing the TAP epitope with the GFP fluorescent fragment. Subsequently, the *cuf2^+^* promoter, up to position −500 from the start codon of the *cuf2^+^* gene, was isolated by PCR using *S. pombe* FY435 genomic DNA as template [48]. Once purified, the DNA fragment was digested with ApaI and PstI and inserted immediately upstream of both the *cuf2* ^+^-*TAP* and *cuf2* ^+^-*GFP* fusion alleles, generating pSK-500*cuf2* ^+^-*TAP* and pSK-500*cuf2* ^+^-*GFP*, respectively. The resulting plasmids were subsequently digested with ApaI and SacI, and the DNA fragments containing the coding sequences of the *cuf2* ^+^-*TAP* and *cuf2* ^+^-*GFP* genes (both under the control of the *cuf2* ^+^ promoter) then inserted into the corresponding sites of both pBP*ade6^+^* and pJK148 [22], [49]. The integrative plasmids were denoted pBP-500*cuf2* ^+^-*TAP*, pBP-500*cuf2* ^+^-*GFP*, pJK-500*cuf2* ^+^-*TAP* and pJK-500*cuf2* ^+^-*GFP*. To monitor the formation of the forespore membrane (FSM) in meiotic cells, an expression plasmid harboring the *GFP-psy1* ^+^ fusion allele was constructed as described previously [37].

### RNA isolation and analysis

Total RNA was extracted using a hot phenol method as described previously [50] and was quantified spectrophotometrically. In the case of the RNase protection assays, 15 µg of RNA per reaction were used as described previously [51]. DNA templates for the antisense riboprobes (Table 2) were cloned into the BamHI and EcoRI sites of the pBluescript SK vector. The resultant constructs were linearized with BamHI for subsequent antisense RNA labelling with [α-^32^P]UTP and T7 RNA polymerase as described previously [51]. *act1^+^* mRNA was probed as an internal control for normalization during quantification of the RNase protection products.

**Table 2:** Riboprobes used to detect steady-state levels of transcripts.

Gene ID	Gene name	Riboprobe length (bp)	Position relative to initiator codon	Source
*SPAC584.02*	*cuf2^+^*	208	+173 to 380	This study
*SPBC32H8.11*	*mei4^+^*	200	+171 to +370	This study
*SPAC1610.03c*	*meu5^+^*	200	+501 to +700	This study
*SPBC1347.03*	*meu14^+^*	210	+351 to +560	This study
*SPAC1B2.03c^+^*	*-*	202	+565 to +766	This study
*SPCC162.04c*	*wtf13^+^*	198	+1222 to +1419	This study
*SPBC1348.01^+^*	*-*	195	+241 to +435	This study
SPBC32H8.12c	*act1^+^*	151	+334 to +485	[51]

### Experimental design and microarray experiments

An experimental design based on the following node, *h^+^/h^+^ pat1-114/pat-1-114 (cuf2^+^/cuf2^+^*) versus *h^+^/h^+^ pat1-114/pat1-114 cuf2Δ/cuf2Δ* was adopted. The meiotic time courses were performed in triplicate. Two of the trials were used in the microarray protocol in which the Cy dyes were swapped. The third trial was used for the analysis of the mRNAs using the RNase protection protocol. After 9 h of meiotic induction under basal conditions, cells corresponding to 5 optical density units (∼1×10^8^ cells/mL) were harvested by centrifugation and snap-frozen by immersion in liquid nitrogen. Total RNA was extracted using a hot phenol method [50]. RNA (20 µg) was labelled by directly incorporating Cy3- and Cy5-dCTP using Superscript (Invitrogen, Carlsbad, CA) reverse transcriptase as described previously [52]. The resulting cDNA preparation was hybridized onto glass DNA microarrays containing probes for 99.3% of all known and predicted *S. pombe* genes. The microarrays were scanned using a GenePix 4000B laser scanner (Axon instruments, Foster City, CA). Data were subsequently analyzed using the GenePix pro software. Unreliable signals were filtered out, and the data were normalized using a customized Per1 script [52]. This script applies cut-off criteria to discard the data from weak signals. Genes that did not yield reproducible results between trials were discarded. Similarly, genes for which 50% of the data points were missing were also discarded. Data acquisition, processing and normalization were performed using the GeneSpring GX software (Agilent Technologies, Cheshire, UK). Normalized signals were exported from GeneSpring into Microsoft Excel spreadsheets and the expression ratios of biological repeat experiments were averaged. A gene was classified as *cuf2^+^*-dependent if its expression was up- or down-regulated ≥1.5-fold in the *cuf2Δ/cuf2Δ* strain, as compared to the wild-type strain grown under basal conditions. Gene annotations were retrieved from GeneDB at the Sanger Institute WEB site http://www.genedb.org/genedb/pombe/index.jsp.

### Protein extraction and Western blot analysis

Whole cell extracts were prepared using a trichloroacetic acid extraction method [53], and equal amounts of each sample were subjected to electrophoresis on 10% sodium dodecyl sulfate-polyacrylamide gels. After electrophoresis, the proteins were electroblotted onto nitrocellulose Hybond-ECL membranes (GE Healthcare, Little Chalfont, Buckinghamshire, UK). The immunoblots were analyzed for the steady-state levels of Cuf2-TAP and the α-tubulin protein using both polyclonal anti-mouse IgG antibody (ICN Biomedicals, Aurora, OH) and monoclonal anti-α-tubulin antibody B-5-1-2 (Sigma-Aldrich Canada, Oakville, ON). After a 1-h incubation with the above-mentioned primary antibodies in 1% powdered skimmed milk in phosphate buffered saline tween (10.1 mM Na_2_HPO_4_, 1.8 mM KH_2_PO_4_, pH 7.4, 138 mM NaCl, 2.7 mM KCl and 0.1% Tween 20), the membranes were washed three times with phosphate buffered saline tween, incubated with the appropriate horseradish peroxidase-conjugated secondary antibodies (GE Healthcare, Little Chalfont, Buckinghamshire, UK) and visualized by chemiluminescence detection on X-ray films.

### Microscopic analysis of Cuf2-GFP localization

*h^+^ cuf2*Δ and *h^−^ cuf2Δ* haploid cells expressing the *cuf2* ^+^-*GFP* allele were grown under low nitrogen conditions and were then crossed in order to produce diploid zygotes. After mating, the cells were quickly transferred to rich YES medium so as to stabilize their diploid state. The azygotic meiosis of diploid cells was synchronously induced by transferring the cells to nitrogen-poor EMM as described previously [37]. At the zero time point when cells had just entered meiosis, they were maintained in nitrogen-poor EMM supplemented with 10 mg/L of adenine, histidine, leucine, uracil and lysine. Culture aliquots were taken up every hour and 5 µg/mL of Hoechst 33342 was added to analyze the progression of meiosis at a single-cell scale. At the indicated meiotic phase, the cells were subjected to microscopic analysis using a 1,000× magnification with the following filters: 465 to 495 nm (GFP) and 340 to 380 nm (Hoechst 33342). Both fluorescence and differential interference contrast images (Nomarski) of the cells were obtained using a Nikon Eclipse E800 epifluorescent microscope (Nikon, Melville, NY) equipped with a Hamamatsu ORCA-ER digital cooled camera (Hamamatsu, Bridgewater, NJ) as described previously [54]. The cell fields shown in this study represent a minimum of five independent experiments. The merged images were obtained using the Simple PCI software version 5.3.0.1102 (Compix, Sewickly, PA).

### Spore viability

*h^+^* wild-type cells and *h^+^* cells harboring a *cuf2Δ* deletion with either an empty integrative vector or a *cuf2^+^-GFP* allele were mated with their corresponding isogenic *h* ^−^ cells (*h^−^* wild-type, *h^−^ cuf2Δ* and *h^−^ cuf2Δ*+*cuf2^+^-GFP*, respectively) onto ME-agar plates. When asci were observed, the ascospores were transferred from ME to YES-agar plates and incubated for 4 h at 36°C so as to accelerate ascus breakdown. More than 200 spores from each of the three strains (*cuf2^+^/cuf2^+^*, *cuf2Δ/cuf2Δ* and *cuf2Δ/cuf2Δ cuf2^+^-GFP*/*cuf2^+^-GFP*) were dissected and sequentially separated approximately 10 mm apart with the aid of a micromanipulator (MS series 200; Singer Instrument, UK). Following spore dissection, the isolated spores were examined to determine whether or not they retain viability on YES-agar plates. Spore viability was expressed as a percentage of the total number of spores dissected. Spore viability counts reported in this study represent a minimum of four independent experiments.

## Supporting Information

Table S1 List of genes that showed elevated expression (averaging ≥1.5-fold) in a cuf2Δ/cuf2Δ mutant (pat1-114/pat1-114 synchronized cells).
(XLS)

Table S2 List of middle-phase meiotic genes that showed elevated expression (averaging ≥1.5-fold) in a cuf2Δ/cuf2Δ mutant (pat1-114/pat1-114 synchronized cells).
(XLS)

Table S3 List of genes that showed elevated expression (averaging ≥1.5-fold) in both cuf2Δ/cuf2Δ and meu5Δ/meu5Δ mutants (pat1-114/pat1-114 synchronized cells).
(XLS)

Table S4 List of genes that showed elevated expression (averaging ≥1.5-fold) in a cuf2Δ/cuf2Δ mutant (pat1-114/pat1-114 synchronized cells).
(XLS)

Table S5 List of genes that show reduced expression in a cuf2Δ/cuf2Δ mutant (pat1-114/pat1-114 synchronized cells).
(XLS)
